# The effect of biomechanical stimulation on osteoblast differentiation of human jaw periosteum-derived stem cells

**DOI:** 10.1186/s40902-017-0104-6

**Published:** 2017-03-05

**Authors:** Ju-Min Lee, Min-Gu Kim, June-Ho Byun, Gyoo-Cheon Kim, Jung-Hoon Ro, Dae-Seok Hwang, Byul-Bora Choi, Geun-Chul Park, Uk-Kyu Kim

**Affiliations:** 1 JUM Oral and Maxillofacial Surgery Clinic, Seoul, South Korea; 2 Department of Oral and Maxillofacial Surgery, School of Medicine and Institute of Health Science, Gyeongsang National University, Jinju, South Korea; 3 Department of Oral Anatomy, School of Dentistry, Pusan National University, Yangsan, South Korea; 4 Department of Biomedical Engineering, School of Medicine, Pusan National Univeristy, Yangsan, South Korea; 5 Department of Oral and Maxillofacial Surgery, School of Dentistry, Pusan National University, Yangsan, South Korea

**Keywords:** Biomechanical stimulation, Cyclic tension force, Osteoblast differentiation, Human jaw periosteum-derived stem cells, New bioreactor, Human mesenchymal stem cells

## Abstract

**Background:**

This study was to investigate the effect of biomechanical stimulation on osteoblast differentiation of human periosteal-derived stem cell using the newly developed bioreactor.

**Methods:**

Human periosteal-derived stem cells were harvested from the mandible during the extraction of an impacted third molar. Using the new bioreactor, 4% cyclic equibiaxial tension force (0.5 Hz) was applied for 2 and 8 h on the stem cells and cultured for 3, 7, and 14 days on the osteogenic medium. Biochemical changes of the osteoblasts after the biomechanical stimulation were investigated. No treatment group was referred to as control group.

**Results:**

Alkaline phosphatase (ALP) activity and ALP messenger RNA (mRNA) expression level were higher in the strain group than those in the control group. The osteocalcin and osteonectin mRNA expressions were higher in the strain group compared to those in the control group on days 7 and 14. The vascular endothelial growth factor (VEGF) mRNA expression was higher in the strain group in comparison to that in the control group. Concentration of alizarin red S corresponding to calcium content was higher in the strain group than in the control group.

**Conclusions:**

The study suggests that cyclic tension force could influence the osteoblast differentiation of periosteal-derived stem cells under optimal stimulation condition and the force could be applicable for tissue engineering.

## Background

In recent years, the need for tissue engineering for new bone formation instead of bone grafting is rapidly increasing in the oral and maxillofacial reconstructive surgery. There are both advantages and disadvantages to tissue engineering. Compared to autogenous bone graft, the biggest advantage of tissue engineering is the elimination of donor site morbidity.

Distraction osteogenesis (DO) is one of the most frequently used tissue engineering-related bone augmentation technique in oral and maxillofacial surgery field. Ever since its original development by Dr. Ilizarov to elongate long bones, DO has been widely used in oral and maxillofacial reconstruction, including alveolar distraction and craniofacial distraction [1, 2]. Many in vitro studies have shown that active bone formation during DO is due to mechanical tension stress that enhances the expression of osteogenesis markers, such as bone morphogenetic protein 2 (BMP-2), BMP-4, osteocalcin, and type I collagen [3, 4].

Although DO has many pros, it is not yet clear how osteoblasts differentiate and matrix formation occurs. In addition, the cons of DO still exist, such as incomplete callus formation, difficult vector control, and relapse [5, 6]. Many studies have attempted to elucidate the mechanism of bone formation in DO and to reduce its side effects. Kim et al. have reported a modified distraction osteogenesis technique, combining tension forces with compression forces to improve a number of the disadvantages associated with DO [7, 8].

To build up viable artificial tissue in laboratory, a biocompatible scaffold consisting of chemical composites seeded with stem cells and growth factors for osteogenesis and angiogenesis are required. In an attempt to overcome limitations of autogenous bone formation, laboratory efforts have been made to grow cells in scaffolds into real tissue. Part of the ongoing research is focused on the development of a dynamic bioreactor which is able to apply biomechanical forces on stem cells within a scaffold in three dimensions.

With support of the Department of Biomedical Engineering at the School of Medicine at Pusan National University, a computer-regulated bioreactor was developed to apply various biomechanical stimulations to cells. Uni- or biaxial strain is generated by selectively controlling the portion of the membrane [9, 10]. Using a dynamic bioreactor in the cell laboratory, tensile strain with various magnitudes and frequency, and combined with compression forces, may provide more suitable circumstances for osteoblast differentiation and proliferation. When the cultured plates that are seeded with cells are subjected to biomechanical stimulation, cells are forced to adapt to the deformation of the culture plate. The responses are expressed as specific biochemical and structural changes. Several studies have reported that biomechanical stimulation influences osteoblast differentiation of human mesenchymal stromal cells (hMSCs) [11, 12]. The jaw periosteum contains osteogenic precursor cells with the potential to differentiate into osteoblasts and chondrocytes. These cells can easily be harvested from human oral cavity [13, 14].

The purpose of this study was to investigate the effect of biomechanical stimulation on osteoblast differentiation of human periosteal-derived stem cells using the newly developed bioreactor. Human periosteal-derived stem cells were harvested from the mandible during the extraction of an impacted third molar. Using the new bioreactor, 4% cyclic equibiaxial tension force (0.5 Hz) was applied for 2 and 8 h on human periosteal-derived stem cells cultured for 3, 7, and 14 days on the osteogenic medium thereafter. Biochemical changes of the osteoblasts after the biomechanical stimulation are investigated in this study.

## Methods

### Reagents

Dulbecco’s modified eagle’s medium (DMEM), fetal bovine serum (FBS), and penicillin/streptomycin were purchased from Gibco (Carlsbad, USA). l-ascorbic acid 2-phosphate, dexamethasone, β-glycerophosphate, alizarin red s, p-nitrophenol, and Alkaline Phosphatase Yellow Liquid Substrate System for ELISA were purchased from Sigma-Aldrich (St. Louis, USA).

### Cell source and culture

Periosteal tissues (5 mm × 20 mm) were harvested from the mandible during surgical extraction of an impacted third molar from patients who had provided the written informed consent, as required by the ethics committee of Gyeongsang National University Hospital. The study was approved by the review board of the institution (Gyeongsang National University Hospital Institutional Review Board, GNUHIRB-2009-057).

Stripped off and washed periosteum was cut into several pieces. Periosteal pieces were placed in a 100-mm culture dish and cultured in DMEM supplemented with 10% FBS, 100 IU/mL penicillin, and 100 mg/mL streptomycin at 37.0 °C in 95% humidified air and 5% CO_2_. Reaching 90% confluence, cells were trypsinized (0.02% trypsin, 0.02% EDTA in PBS) for 5 min, centrifuged for 5 min at 1500 rpm, and expanded. After passage 3, the periosteal cells were trypsinized and subsequently suspended in the osteogenic induction in DMEM medium supplemented with 10% fetal bovine serum, 50 mg/mL L-ascorbic acid 2-phosphate (Sigma-Aldrich, St. Louis, USA), 10 nM dexamethasone (Sigma-Aldrich, St. Louis, USA), and 10 mM β-glycerophosphate (Sigma-Aldrich, St. Louis, USA) at a density of 3 × 10^4^ cells/well in a collagen coated 6-well plate. The medium was changed every 3 days during the incubation period.

### Design and characteristics of new bioreactor, mechanical stimulation

**Fig. 1:**
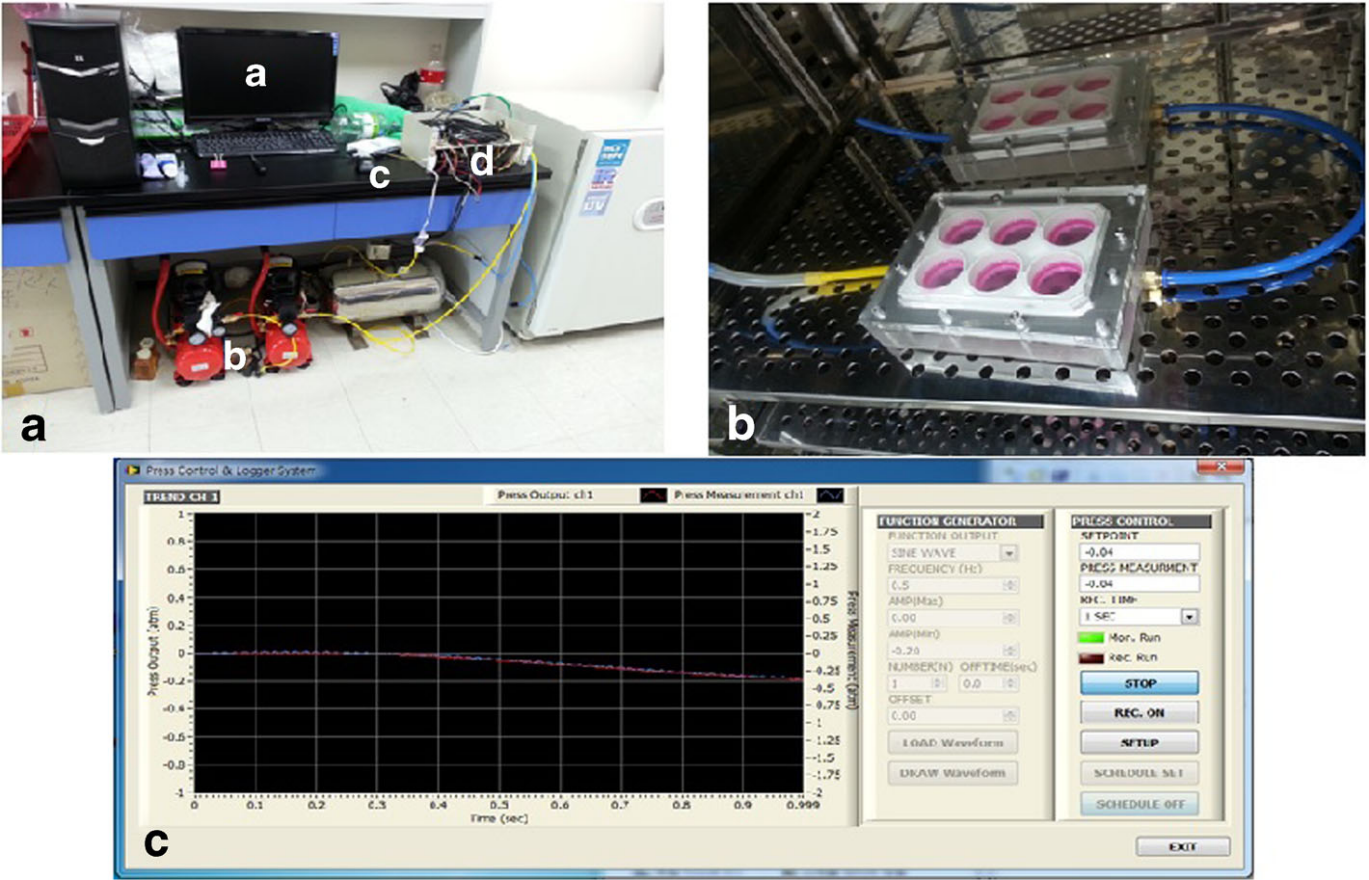
New bioreactor (**a** Photographs of New Bioreactor. a: computer monitor, b: vacuum pump and compressors, c: bioreactor, d: pressure control tower, **b** Tensile strain Inducing Cell Culture Plate inside a standard incubator, **c** The screen capture of the waveform generator program coded in LabVIEW^TM^. NI USB-6215 DAQ board is used for the interface)

New bioreactor which can generate cyclic tension strain was implemented by modifying the Flexcell™ system in cooperation with the Department of Biomedical Engineering, School of Medicine, at Pusan National University. The system consists of a waveform generator which sends electronic signal of arbitrary functions out and a pneumatic controller which sends the air pressure waveform out corresponding to the input electronic signal through a feedback system. A compressor and a vacuum pump were used to supply 7 ~ 8 bar compressed air as well as −1 bar vacuum pressure to the proportional valve (VP50, Norgren, UK). A pressure regulator (RS-4, Fujikura, Tokyo, Japan) was used to supply a stable 6-bar pressure to the valve. A pressure sensor (Auto transducer, Ace medical, Korea), which is identical to an invasive blood pressure monitoring device, is attached to the cell culture plates (BioFlex® Culture Plates, Flexcell® international corp., Hillsborough, USA) and used to measure the real pressure applied to the plates (Fig. 1).

Reaching 100% confluence, cultured periosteal cells were transferred to the osteogenic induction medium. The plates were then placed into the new bioreactor in which the 6-well plates are docked. A pump and a pressure reservoir generated vacuum waveforms to deform the wells and stretch the silicone membranes. The membranes with the cells were subjected to different levels of cyclic equibiaxial mechanical strain of 4% strain at 0.5 Hz for 2 and 8 h. Tension force was measured by real-time image processing with a web camera that feeds back into the pressure controller. The range of tensile strain to deform the culture plate was measured as 4% with the settings. This condition of biomechanical stimulation was defined through several preliminary cell studies on frequency and magnitude of mechanical strain. These preliminary studies are supposed be separately reported in another thesis.

### Qualitative staining of alkaline phosphatase (ALP)

**Fig. 2:**
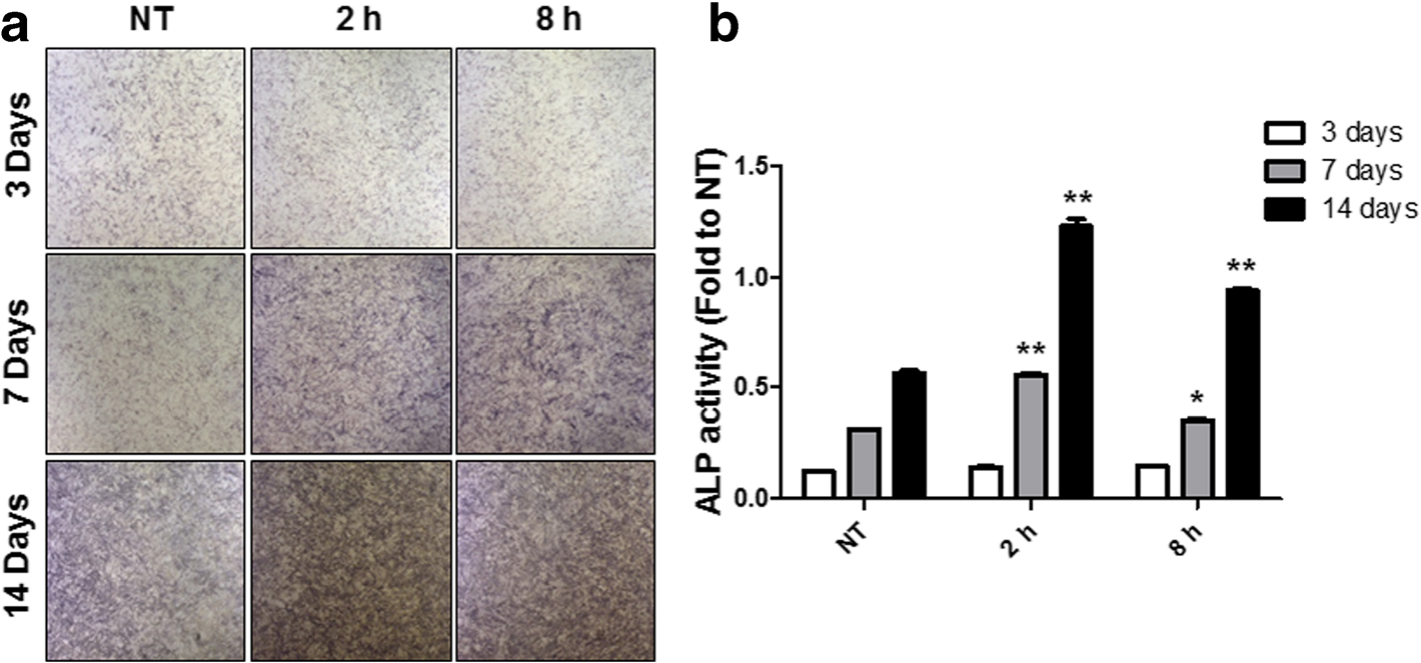
Effect of cyclic equibiaxial tension on human periosteal-derived stem cell differentiation. **a** ALP was stained by BCIP/NBT. **b** ALP activity was measured by flow cytometry. The statistical significance of each group were analyzed by the paired *t* test (*p < 0.05 and **p < 0.01)

Reaching 100% confluence, cultured periosteal cells were transferred to the osteogenic induction medium. The cells were subjected to different levels of cyclic equibiaxial mechanical strain. Periosteal-derived stem cells were incubated for 3, 7, and 14 days. After rinsing monolayer cells with PBS, the cells were fixed in 3.7% formaldehyde and 90% ethanol solution for 2 min and washed in Tris buffered saline (TBS) for 10 min. Then, the cells were stained with fast 5-bromo-4-chloro-3-indolyl phosphate and nitroblue tetrazolium (BCIP/NBT) alkaline phophatase substrate (Amresco, Ohio, USA) for 10 min in the dark at room temperature. The reaction was stopped by removing the substrate solution and washed with distilled water. ALP-positive cells can be visualized in dark purple color under the stereoscopic microscope (Olympus SZX10, Olympus, Tokyo, JAPAN) (Fig. 2).

### Quantitative measurement of ALP activity

Reaching 100% confluence, cultured periosteal cells were transferred to the osteogenic induction medium. The cells were subjected to different levels of cyclic equibiaxial mechanical strain. Periosteal-derived stem cells were incubated for 3, 7, and 14 days. The cultured cells were washed twice with PBS and collected in 0.1 mol/l Tris buffer containing 0.1% Triton X-100. Cellular membranes were lysed by a freeze–thaw method in cell lysis solution (0.2% Triton X-100, 10 mM Tris (pH 7.0), and 1 mM EDTA). Aliquots of supernatants were subjected to ALP activity measurement and protein assay according to Bradford’s method. The ALP activity was determined using 50 mmol/l p-nitrophenylphosphate in a sodium carbonate buffer at pH 10.4, followed by incubation at 37 °C for 30 min. After adding 0.2 M NaOH, the amount of released p-nitrophenylphosphate was estimated by measuring the absorbance at 405 nm by flow cytometry (Fig. 2).

### Alizarin red S staining

Reaching 100% confluence, cultured periosteal cells were transferred to the osteogenic induction medium. The cells were subjected to different levels of cyclic equibiaxial mechanical strain. Periosteal-derived stem cells were incubated for 14 days. The cells were rinsed with phosphate-buffered saline (PBS) and fixed in 10% formaldehyde in PBS for 15 min. The cells were washed with distilled water, treated with a 2% alizarin red S solution (pH 4.5) for 10 min, and washed several times with distilled water to remove the remaining stain. Calcium deposits can be visualized by their red color. To quantify the staining, cultured cells were destained using 10% cetylpyridinium chloride (CPC) in 10 mM sodium phosphate, pH 7.0, for 2 h. Aliquots of exacts were diluted 10-fold in 10% CPC solution, and alizarin red S concentration was determined by absorbance measurement in form of optical density (OD) at 570 nm wavelength on a multiplate ELISA reader(Thermo-Lab systems, Leuven, Belgium). Microscopic images were taken using Olympus CKX40 (Olympus, Tokyo, Japan) with magnification of ×40. Periosteal-derived cells staining red represent positive alizarin red S staining.

### Measurement of calcium content

Reaching 100% confluence, cultured periosteal cells were transferred to the osteogenic induction medium. The cells were subjected to different levels of cyclic equibiaxial mechanical strain. Periosteal-derived stem cells were incubated for 14 days and rinsed off with PBS. Periosteal-derived cells were decalcified with 0.6 N HCl over a period of 24 h. The calcium content of the supernatants was determined by spectrophotometry using the o-cresolphthalein method (Calcium C-test Wako, Wako Pure Chemical Industries, Osaka, Japan). After decalcification, total protein content in the supernatants was quantitatively measured using a calcium colorimetric assay kit (Pierce Chemical Co, Rockford, USA).

### Total RNA extraction, RT-PCR of ALP, osteocalcin (OC), osteonectin (ON), osteopontin (OP), and vascular endothelial growth factor (VEGF)

Total RNA was extracted from each sample using the Trizol reagents (Invitrogen) according to the manufacturer’s instructions and treated with DNase I (Promega, Madison, WI). First strand complementary DNA was reverse-transcribed using the Maxime RT PreMix kit (iNtRON, Korea). The PCR products were resolved by electrophoresis on 1.5% ethidium bromide-stained agarose gel. Quantifications of ALP, OC, ON, OP, and VEGF are expressed as relative mRNA levels of genes visualized by luminescence LAS (FUJIFILM, Tokyo, Japan). Detailed information of primers used in this study is shown in Table 1.

**Table 1:** Detailed information of primers

Gene name	Sequence	Product size (bp)	Tm (°C)
Osteocalcin	AGAGACCCAGGCGCTACCT	259	62.3
	CTGGGAGGTCAGGGCAAG		59.33
Osteopontin	GGTCACTGATTTTCCCACGG	274	58.83
	GTCCTTCCCACGGCTGTC		60.05
Osteonectin	TGGAGGCAGGAGACCACC	271	60.61
	TCCTTCTGCTTGATGCCG		57.38
VEGF	CCACCATGCCAAGTGGTC	307	58.31
	CATCTCTCCTATGTGCTGGC		57.83
Alkaline phosphatase-1	TTTGGTGGATACACCCCC	176	56.05
	GCCTGGTAGTTGTTGTGAGC		59.12
GAPDH	ACTGGCATGGCCTTCCGT	289	61.65
	CCACCCTGTTGCTGTAGCC		60.68

### Statistical analysis

All experiments were paired with their controls. Data between groups were analyzed by the paired *t* test using SPSS 12.0 statistical software program for windows (SPSS Inc., Chicago, USA). Probability of null hypothesis <5% (*p* < 0.05) was considered statistically significant.

## Results

### Analysis of ALP expression

Human periosteal-derived cells were cultured in osteogenic conditions for 3, 7, and 14 days. After ALP staining by BCIP/NBT, the expression was observed using phase contrast microscope (Fig. 2a). ALP-positive periosteal-derived cells are indicated by blue or purple staining. Periosteal-derived cells showed weak positive ALP expression at day 3 of incubation. The expressions of ALP increased up to 14 day time dependently. The strain (2 and 8 h) group showed higher level of ALP expression than the no treatment group.

### Quantitative measurement of ALP activity

The ALP activity of the periosteal-derived cells was notably higher in the strain (2 and 8 h) group compared to that in the no treatment group (Fig. 2b). The levels of ALP activity of the periosteal-derived cells in the no treatment group and strain group (2 and 8 h) on day 3 were 0.0251, 0.0424, and 0.0464, respectively. On day 7, the ALP activity levels in the no treatment group and strain group (2 and 8 h) were 0.3085, 0.5637, and 0.3566, respectively. On day 14, the levels of ALP activity of the cells in the no treatment group and strain group (2 and 8 h) were 0.5822, 1.2911, and 0.9362, respectively. Strain group (2 and 8 h) showed a 1.85-fold increase on day 3 (8 h), 1.82-fold increase on day 7 (2 h), and 2.22-fold increase on day 14 (2 h) compared with no treatment group (Fig. 2b).

### Alizarin red S staining and quantitative measurement of calcium content

**Fig. 3:**
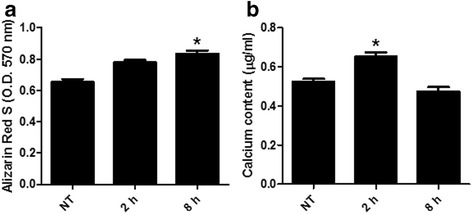
Quantification of alizarion red S staining (**a**) and calcium (**b**) by cyclic equibiaxial tension in periosteal-derived cells. The statistical significance of each groups were analyzed by the paired *t* test (*p < 0.05)

Periosteal-derived cells stained red represented positive alizarin red S staining. As shown in Fig. 3a, alizarin red S-positive mineralization deposits were observed grossly as sparse red crystals on day 14 of incubation. Absorbance measurement found a higher concentration of alizarin red S in strain groups compared to that in the no treatment group. As shown in Fig. 3b, calcium content was significantly higher in the 2 h strain group (0.6113 μg/ml) than that in the no treatment group (0.5529 μg/ml) and 8 h strain group (0.4966 μg/ml).

### RT-PCR

**Fig. 4:**
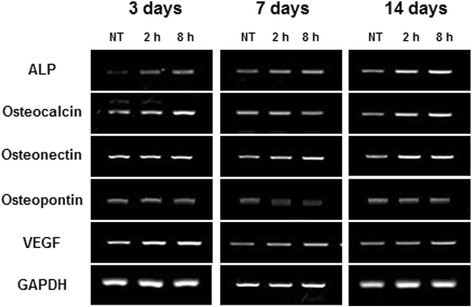
The mRNA expression of ALP, osteocalcin, osteonectin, osteopontin, and VEGF in the periosteal-derived stem cells

**Fig. 5:**
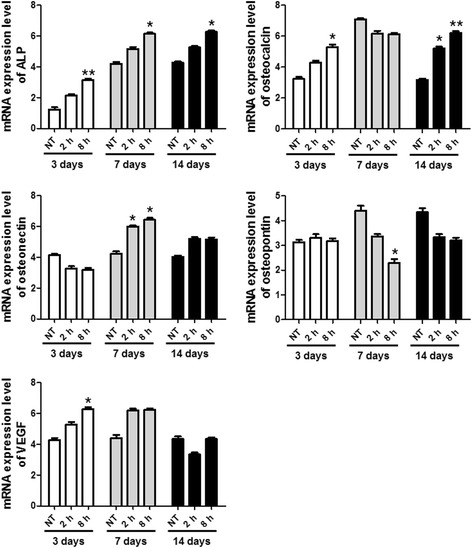
Quantification of mRNA expression of ALP, osteocalcin, osteonectin, osteopontin, and VEGF in the periosteal-derived stem cells. The statistical significance of each group were analyzed by the paired *t* test (*p < 0.05 and **p < 0.01)

No treatment group was used as negative control. As shown in Figs. 4 and 5, the mRNA level of ALP in the periosteal-derived cells was higher in the mechanical strain group compared to that in the no treatment group. The ALP expression first appeared on day 3 and then increased on days 7 and 14. The expression level of osteocalcin mRNA in the periosteal-derived stem cells was also higher in the strain group (8 h > 2 h) than in the no treatment group. The osteocalcin expressions appeared on day 3 and decreased at day 7. On day 7, a remarkable difference in osteocalcin expression was not observed between the strain group (2 and 8 h) and the control group. On day 14, osteocalcin level of the strain group was higher than that of the control group. The mRNA expression of VEGF in the strain group was higher than that in the no treatment group on days 3 and 7, but the strain group did not show higher levels of VEGF expression compared to the control group on day 14. Osteopontin mRNA expression in the strain group was similar to that in the control group on day 3. However, osteopontin mRNA expression in the control group was relatively higher in comparison to that in the strain group on days 7 and 14. The mRNA expression of osteonectin was lower in the strain group on day 3 but became higher than the no treatment group on days 7 and 14. Osteonectin mRNA expression was constant in the no treatment group. Quantification of genes was expressed as relative mRNA levels by densitometry (Fig. 5). The intensity of the bands in the no treatment group on day 3 was regarded as 1. Glyceraldehyde 3-phosphate dehydrogenase (GAPDH) was used as loading control, and detailed information of primers are shown in Table 1.

## Discussion

For this study, a new computer-regulated bioreactor was manufactured by modifying the Flexcell™ system in conjunction with the Department of Biomedical Engineering at Pusan National University. The pressure control was regulated by a pressure regulator (RS-4, Fujikura, Japan) and a pressure sensor (Auto transducer, Ace medical, Korea). The pressure regulator reduces 1000 KPa of compressed air into 14 ~ 420 KPa. The pressure sensor converts the pressure of 100 mmHg to an electric signal of 1 V and is identical to the sensor used for measuring arterial blood pressure. Thus, biomechanical stimulation with various frequency and magnitude can be applied.

The cells were subjected to different levels of cyclic equibiaxial mechanical strain. The biomechanical stimulation of 4% strain at 0.5 Hz for 2 and 8 h was set up after preliminary studies on ALP activity and calcium content at various frequencies (from 0.4 to 3 MHz) and magnitudes (from 1 to 10%) of mechanical strain.

There is a set of protein markers including type I collagen, alkaline phosphatase (ALP), transforming growth factor β1 (TGF-β1), vascular endothelial growth factor (VEGF), nitric oxide (NO), BMP-2, BMP-4, BMP-6, BMP-7, osteonectin, and osteocalcin that are expressed during the differentiation of osteoprogenitor cells into osteoblasts [11, 15, 16].

Generally, ALP is considered to be an early marker of osteoblast differentiation. As ALP is a byproduct of osteoblast activity, increased level of ALP refers to active bone formation. The present study examined the osteogenic activity of cultured human periosteal-derived stem cells. ALP expression and calcium deposit were used as early and late markers for osteogenesis and were detected by p-nitrophenylphosphate staining and alizarin red staining, respectively. The ALP activity was evaluated qualitatively and quantitatively during osteoblast differentiation. ALP-positive human periosteal-derived stem cells are identified by blue/purple staining. On day 3 of incubation following exposure to strain, the periosteal-derived stem cells showed a weak expression of ALP and were allowed to grow for up to 14 days. About twofold higher ALP activity was observed in the periosteal-derived stem cells exposed to biomechanical strain compared to the control group. Additionally, the ALP activity increased in a time-dependent manner up to 14 days of incubation.

RT-PCR was performed to investigate the mRNA expressions of proteins related to bone formation. The expression of ALP mRNA showed a similar trend to that of ALP activity in that the ALP mRNA expression levels were higher in the periosteal-derived stem cells exposed to biomechanical strain compared to those in the periosteal-derived stem cells in the control group.

Osteocalcin is a noncollagenous protein found in bone and dentin. Osteocalcin is secreted by osteoblasts and is related to matrix mineralization and calcium ion homeostasis. Its secretion is associated with the late phase of osteoblast differentiation. In this study, the periosteal-derived stem cells exposed to biomechanical strain expressed higher levels of osteocalcin mRNA on days 7 and 14 compared to the periosteal-derived stem cells in the control group.

VEGF is a signal protein that stimulates vasculogenesis and angiogenesis. The VEGF expression correlates with the differentiation of the cultured periosteal-derived stem cells. An increased VEGF secretion from cultured human periosteal-derived stem cells occurs during periosteal osteogenesis. While the mRNA expression of VEGF in the human periosteal-derived stem cells exposed to biomechanical strain was higher compared to that in the control group on days 3 and 7, it decreased on day 14.

Osteopontin is an important factor in bone remodeling, playing a role in anchoring osteoclasts to the mineral matrix of the bones [17]. Osteopontin expression in the bone occurs in osteoblasts, osteocytes, as well as osteoclasts. It binds to calcium ions and acts as a mineralization inhibitor to regulate crystal growth. Osteonectin is a glycoprotein that binds calcium, and its secretion by osteoblasts initiates mineralization and promotes mineral crystal formation. In addition to bone mineral calcium, osteonectin also shows affinity for collagen. In this study, osteopontin mRNA expression levels in the periosteal-derived stem cells exposed to biomechanical strain were similar to those of the control group on day 3. However, osteopontin mRNA expression in the control group was relatively higher than that in the strain group on days 7 and 14. In contrast, the mRNA expression of osteonectin was lower in the strain group compared to that in the control group on day 3, while it increased on days 7 and 14. The expression of osteonectin mRNA was constant in the control group. Based on these RT-PCR results, the biomechanical strain induced a relatively high mRNA levels of ALP (days 3, 7, and 14), osteocalcin (days 7 and 14), osteonectin, and VEGF (days 3 and 7) in the human periosteal-derived stem cells, suggesting that tension force has positive effect on osteoblasts differentiation.

In contrast to ALP, osteocalcin, osteonectin, and VEGF, the tension forces did not appear to significantly affect mRNA expression of osteopontin. Further studies are needed to determine the role of osteopontin in osteoblast differentiation.

The jaw periosteum is one of the sources of osteogenic precursor cells for osteogenesis. Like bone marrow-derived mesenchymal stem cells, periosteal-derived stem cells are multipotent and are able to differentiate into osteoblasts and chondrocytes [18]. The major advantage of the periosteal-derived stem cells is the ease of harvesting compared to that of the more widely used bone marrow-derived stem cells in the oral and maxillofacial surgery [19–21]. Small pieces of periosteum can be easily obtained from various intraoral procedures.

Mineralization of bone cells can be assessed by fluorescent calcein binding, Von Kossa staining, and alizarin red S staining [22, 23]. Alizarin red S staining is one of the most common methods used to examine a mineralized matrix [24, 25]. Both Von Kossa and alizarin red S staining allow simultaneous evaluation of mineral distribution and inspection of small structures by phase contrast microscope. Alizarin red S staining is particularly versatile in that the dye can be extracted from the stained monolayer and readily quantified. In this study, alizarin red S-positive mineralization was first observed at 2 h in the strain-exposed human periosteal-derived stem cells on day 14. Based on the absorbance measurement at OD 570 nm, the concentration of alizarin red S was highest at 2 h in the strain-exposed periosteal-derived stem cells. Similarly, the calcium content was significantly higher at 2 and 8 h in the strain-exposed periosteal-derived stem cells than in the control group. Alizarin red S staining and calcium content analysis show that human periosteal-derived stem cells were differentiated into osteoblasts and involved in the matrix mineralization.

## Conclusions

These results suggest that the human periosteal-derived stem cells could be regarded as active periosteal-derived osteoblasts and cyclic tension forces at low frequency and low magnitude have a positive influence on osteoblast differentiation of human periosteal-derived stem cells in vitro. These findings could be used in future studies on optimal conditions for biomechanical stimulation. Currently, the effect of cyclic compression forces and combination of cyclic tension on human periosteal-derived stem cells is being investigated as a step towards for more effective biomechanical stimulation. Further studies are needed to elucidate the optimal force frequency and magnitude for improved osteoblast differentiation for tissue engineering and distraction osteogenesis treatment.

## Abbreviations

ALP: Alkaline phosphatase
BMP: Bone morphogenetic protein
CPC: Cetylpyridinium chloride
DMEM: Dulbecco’s modified eagle’s medium
DO: Distraction osteogenesis
FBS: Fetal bovine serum
hMSCs: Human mesenchymal stromal cells
OC: Osteocalcin
OD: Optical density
ON: Osteonectin
OP: Osteopontin
TGF-β1: Transforming growth factor β1
VEGF: Vascular endothelial growth factor

## References

[1] Ilizarov G (1971). Basic principles of transosseous compression and distraction osteosynthesis. Ortopediia travmatologiia i protezirovanie.

[2] Ilizarov GA (1989). The tension-stress effect on the genesis and growth of tissues: Part I. The influence of stability of fixation and soft-tissue preservation. Clin Orthop Relat Res.

[3] Ignatius A, Blessing H, Liedert A, Schmidt C, Neidlinger-Wilke C, Kaspar D (2005). Tissue engineering of bone: effects of mechanical strain on osteoblastic cells in type I collagen matrices. Biomaterials.

[4] Tanaka SM, Li J, Duncan RL, Yokota H, Burr DB, Turner CH (2003). Effects of broad frequency vibration on cultured osteoblasts. J Biomech.

[5] Mofid MM, Manson PN, Robertson BC, Tufaro AP, Elias JJ, Vander Kolk CA (2001). Craniofacial distraction osteogenesis: a review of 3278 cases. Plast Reconstr Surg.

[6] Garcia AG, Martin MS, Vila PG, Maceiras JL (2002). Minor complications arising in alveolar distraction osteogenesis. J Oral Maxillofac Surg.

[7] Kim U-K, Chung I-K, Lee K-H, Swift JQ, Seong W-J, Ko C-C (2006). Bone regeneration in mandibular distraction osteogenesis combined with compression stimulation. J Oral Maxillofac Surg.

[8] Kim U-K, Park S-J, Seong W-J, Heo J, Hwang D-S, Kim Y-D (2010). Expression of TGF-β1, osteonectin, and BMP-4 in mandibular distraction osteogenesis with compression stimulation: reverse transcriptase-polymerase chain reaction study and biomechanical test. J Oral Maxillofac Surg.

[9] Hamilton DW, Maul TM, Vorp DA (2004). Characterization of the response of bone marrow-derived progenitor cells to cyclic strain: implications for vascular tissue-engineering applications. Tissue Eng.

[10] Matheson LA, Maksym GN, Santerre JP, Labow RS (2007). Differential effects of uniaxial and biaxial strain on U937 macrophage-like cell morphology: influence of extracellular matrix type proteins. J Biomed Mater Res Part A.

[11] Kim I, Song Y, Lee B, Hwang S (2012). Human mesenchymal stromal cells are mechanosensitive to vibration stimuli. J Dent Res.

[12] Wu Y, Zhang X, Zhang P, Fang B, Jiang L (2012). Intermittent traction stretch promotes the osteoblastic differentiation of bone mesenchymal stem cells by the ERK1/2-activated Cbfa1 pathway. Connect Tissue Res.

[13] Breitbart AS, Grande DA, Kessler R, Ryaby JT, Fitzsimmons RJ, Grant RT (1998). Tissue engineered bone repair of calvarial defects using cultured periosteal cells. Plast Reconstr Surg.

[14] Hutmacher DW, Sittinger M (2003). Periosteal cells in bone tissue engineering. Tissue Eng.

[15] Bhatt KA, Chang EI, Warren SM, Lin S-e, Bastidas N, Ghali S (2007). Uniaxial mechanical strain: an in vitro correlate to distraction osteogenesis. J Surg Res.

[16] Hara F, Fukuda K, Ueno M, Hamanishi C, Tanaka S (1999). Pertussis toxin-sensitive G proteins as mediators of stretch-induced decrease in nitric-oxide release of osteoblast-like cells. J Orthop Res.

[17] Choi S, Kim J, Kang E-J, Lee S-W, Park M-C, Park Y-B (2008). Osteopontin might be involved in bone remodelling rather than in inflammation in ankylosing spondylitis. Rheumatology.

[18] Park B-W, Hah Y-S, Kim DR, Kim J-R, Byun J-H (2007). Osteogenic phenotypes and mineralization of cultured human periosteal-derived cells. Arch Oral Biol.

[19] Jaiswal N, Haynesworth SE, Caplan AI, Bruder SP (1997). Osteogenic differentiation of purified, culture-expanded human mesenchymal stem cells in vitro. J Cell Biochem.

[20] Kotobuki N, Hirose M, Machida H, Katou Y, Muraki K, Takakura Y (2005). Viability and osteogenic potential of cryopreserved human bone marrow-derived mesenchymal cells. Tissue Eng.

[21] Park B-W, Hah Y-S, Kim DR, Kim J-R, Byun J-H (2008). Vascular endothelial growth factor expression in cultured periosteal-derived cells. Oral Surg Oral Med Oral Pathol Oral Radiol Endod.

[22] Hale L, Ma Y, Santerre R (2000). Semi-quantitative fluorescence analysis of calcein binding as a measurement of in vitro mineralization. Calcif Tissue Int.

[23] Anselme K, Broux O, Noel B, Bouxin B, Bascoulergue G, Dudermel A-F (2002). In vitro control of human bone marrow stromal cells for bone tissue engineering. Tissue Eng.

[24] Kim H, Iwasaki K, Miyake T, Shiozawa T, Nozaki S, Yajima K (2003). Changes in bone turnover markers during 14-day 6 head-down bed rest. J Bone Miner Metab.

[25] Deckers MM, Karperien M, van der Bent C, Yamashita T, Papapoulos SE, Löwik CW (2000). Expression of vascular endothelial growth factors and their receptors during osteoblast differentiation. Endocrinology.

